# Comparative venom gland transcriptomics of *Naja kaouthia* (monocled cobra) from Malaysia and Thailand: elucidating geographical venom variation and insights into sequence novelty

**DOI:** 10.7717/peerj.3142

**Published:** 2017-04-05

**Authors:** Kae Yi Tan, Choo Hock Tan, Lawan Chanhome, Nget Hong Tan

**Affiliations:** 1 Department of Molecular Medicine, Faculty of Medicine, University of Malaya, Kuala Lumpur, Malaysia; 2 Department of Pharmacology, Faculty of Medicine, University of Malaya, Kuala Lumpur, Malaysia; 3 Queen Saovabha Memorial Institute, Bangkok, Thailand

**Keywords:** Venom gland transcriptomics, *Naja kaouthia*, Monocled cobra, Three-finger toxins, Toxin sequence, Snake venom, Geographical variation

## Abstract

**Background:**

The monocled cobra (*Naja kaouthia*) is a medically important venomous snake in Southeast Asia. Its venom has been shown to vary geographically in relation to venom composition and neurotoxic activity, indicating vast diversity of the toxin genes within the species. To investigate the polygenic trait of the venom and its locale-specific variation, we profiled and compared the venom gland transcriptomes of *N. kaouthia* from Malaysia (NK-M) and Thailand (NK-T) applying next-generation sequencing (NGS) technology.

**Methods:**

The transcriptomes were sequenced on the Illumina HiSeq platform, assembled and followed by transcript clustering and annotations for gene expression and function. Pairwise or multiple sequence alignments were conducted on the toxin genes expressed. Substitution rates were studied for the major toxins co-expressed in NK-M and NK-T.

**Results and discussion:**

The toxin transcripts showed high redundancy (41–82% of the total mRNA expression) and comprised 23 gene families expressed in NK-M and NK-T, respectively (22 gene families were co-expressed). Among the venom genes, three-finger toxins (3FTxs) predominated in the expression, with multiple sequences noted. Comparative analysis and selection study revealed that 3FTxs are genetically conserved between the geographical specimens whilst demonstrating distinct differential expression patterns, implying gene up-regulation for selected principal toxins, or alternatively, enhanced transcript degradation or lack of transcription of certain traits. One of the striking features that elucidates the inter-geographical venom variation is the up-regulation of α-neurotoxins (constitutes ∼80.0% of toxin’s fragments per kilobase of exon model per million mapped reads (FPKM)), particularly the long-chain α-elapitoxin-Nk2a (48.3%) in NK-T but only 1.7% was noted in NK-M. Instead, short neurotoxin isoforms were up-regulated in NK-M (46.4%). Another distinct transcriptional pattern observed is the exclusively and abundantly expressed cytotoxin CTX-3 in NK-T. The findings suggested correlation with the geographical variation in proteome and toxicity of the venom, and support the call for optimising antivenom production and use in the region. Besides, the current study uncovered full and partial sequences of numerous toxin genes from *N. kaouthia* which have not been reported hitherto; these include *N. kaouthia*-specific l-amino acid oxidase (LAAO), snake venom serine protease (SVSP), cystatin, acetylcholinesterase (AChE), hyaluronidase (HYA), waprin, phospholipase B (PLB), aminopeptidase (AP), neprilysin, etc. Taken together, the findings further enrich the snake toxin database and provide deeper insights into the genetic diversity of cobra venom toxins.

## Introduction

Snake venoms consist mainly of pharmacologically active components fine-tuned by evolution against the physiological processes that maintain prey homeostasis (Calvete et al., 2009; Casewell et al., 2013). Positive selection and repeated duplication have been implicated in toxin genes of many lineages, reflecting the adaptive contribution of snake venoms to fitness, and as a source of selective pressure that drives the predator–prey ‘arms race’ coevolution (Margres et al., 2013). However, the origin of toxin genes has been disputable and different mechanisms are available to explain the evolution of snake venom (Casewell et al., 2014; Hargreaves et al., 2014; Reyes-Velasco et al., 2015). A common perspective holds that the duplication of genes generating paralogous give rise to multigene families following the ‘birth and death’ mode of evolution; where some gene copies went through extensive neofunctionalisation at accelerated rates, while the non-functional forms are gradually lost through degradation or transformed into pseudogenes, a process of purifying selection that further preserves the useful toxin arsenal (Casewell et al., 2013; Nei, Gu & Sitnikova, 1997; Sunagar et al., 2013; Sunagar & Moran, 2015). The long evolutionary processes give rise to the extreme complexity of snake venom, an ecologically critical phenotype of venomous snakes which also ramifies into the treatment management of snakebite envenomation.

To date, snakebite envenomation remains a serious public health concern in many developing and underdeveloped nations (Warrell, 2010). It is not only an occupational hazard for the agricultural population but also becoming a public health issue due to the encroaching of human activities into the habitat of venomous snakes (Jamaiah et al., 2006). In Southeast Asia, cobra (*Naja* sp.) is a common cause of snakebites with life-threatening outcomes: cobras are capable of injecting large amount of venom and the venom generally contains potent neurotoxins that can paralyse the envenomed subjects in minutes (Tan & Tan, 2015). Listed under category I of medically important venomous snakes (Chippaux, 2010), the monocled cobra (*Naja kaouthia*) is a widespread species found across the Eastern Indian subcontinent to most parts of Indochina (including the Peninsular Malaya) and the southern part of China. Because of the vast geographical distribution and the morphological variety, this species was previously given different names in various geographical areas, resulting in a period of confusion whereby cobras in Southeast Asia were often indiscriminately labelled as *Naja naja kaouthia*, *N. naja sputatrix*, *N. naja siamensis*, *N. naja atra*, etc. The current systematics based on molecular phylogenetics has resolved the issue and clearly regarded the species of monocled cobra as *N. kaouthia* (Wüster, 1996). The venom proteome of *N. kaouthia* had also been studied to some extents over the years (Kulkeaw et al., 2007; Laustsen et al., 2015; Vejayan, Khoon & Ibrahim, 2014). It is noteworthy that a recent study has provided a global comparison on the proteomic details of *N. kaouthia* venoms from three different Southeast Asian regions (Malaysia, Thailand and Vietnam) qualitatively (profiling protein subtypes) and quantitatively (addressing relative abundances of the proteins) (Tan et al., 2015d). Substantial venom variations were noted across the geographical samples; notably the Thai *N. kaouthia* venom contains a much higher amount of long-chain neurotoxin (LNTX, ∼33%) as compared to the Malaysian (∼4%) and the Vietnamese specimens (not detected), respectively. The proteomic variation was further demonstrated at functional levels through studies on venom lethality, neuromuscular depressant activity and immunological neutralisation using antivenom (Tan et al., 2016a, 2016b). The molecular diversity and the genetic variability of the toxins in the venom, however have not been comprehensively investigated.

To gain a deeper insight into the geographical variation of *N. kaouthia* venom, we applied a comparative transcriptomic approach of the venom glands to delineate the genetic variability of the cobra. De novo assembled venom gland transcriptomes of *N. kaouthia* from Malaysia (NK-M) and Thailand (NK-T) were investigated (the transcriptomes are natural features) using next-generation sequencing (NGS) technology at the Illumina platform, imparting a paired-end approach as previously described (Aird et al., 2013; Rokyta et al., 2012; Tan et al., 2015a). It is hoped that by correlating the transcriptomic findings to its proteome and to the biological activities of the venom, the study will propel the understanding of the spectrum and the molecular diversity of the venom genes in this species.

## Methods

### Snake venom gland preparation

The adult *N. kaouthia* specimen from Malaysia was captured from the northern region of Peninsular Malaya and the specimen from Thailand was captured from the southern region near Bangkok, Thailand. Snake venom milking was carried out prior to tissue harvesting to stimulate the transcription process, and the snake was allowed to rest for four days to maximise the transcription (Rotenberg, Bamberger & Kochva, 1971). Following euthanasia, the venom glands were swiftly removed and sectioned into dimensions of < 5×5 mm before preserving them in a RNAlater® (Ambion, Texas, USA) solution at a 1:10 volume ratio. The solution was allowed to permeate the tissues at 4 °C overnight before transferring to −80 °C for storage until further use. The tissue collection for research purposes was conducted in accordance with the protocol and guidelines approved by the Institutional Animal Use and Care Committee (IACUC) of the University of Malaya, Malaysia (approval number: #2013-11-12/PHAR/R/TCH).

### Total RNA extraction and mRNA purification

The dissected venom gland tissues were submerged and homogenised in a 1 ml glass homogeniser with TRIzol solution (Invitrogen, Carlsbad, CA, USA) under sterile conditions. This was followed by the addition of 20% chloroform, centrifugation and RNA-free DNAase I treatment to separate RNA from cellular debris and residual DNA. The isolated RNA was then pelleted with isopropyl alcohol and washed with 75% ethanol. Polyadenylated mRNA (poly(A)^+^ mRNA) was subsequently purified from 20 mg of total RNA using oligo (dT) magnetic beads as per the (Illumina, San Diego, CA, USA) manufacturer’s instructions. Two rounds of poly(A)^+^ mRNA isolation were performed.

### cDNA library construction and sequencing

Enriched poly(A)^+^ mRNA isolated from the total venom gland RNA was used for cDNA construction. Following purification, the mRNA isolated was fragmented in standard buffers containing divalent cations (Zn^2+^) into short fragments, which acted as templates for cDNA synthesis. Random hexamer-primer (*N6*) was used to synthesise the first-strand cDNA, followed by second-strand cDNA synthesis with the double-stranded cDNA as input material, using second strand buffers, dNTPs, RNase H and DNA polymerase I. Short fragments were purified with QIAquick PCR extraction kit (Qiagen, Valencia, CA, USA) and resolved with *EB* buffer for end repair and the addition of single adenine nucleotide to aid in the subsequent ligation of the Illumina adaptors, which contain a single thymine (T) base overhang at their 3′ ends. Following the ligation of sequencing adaptors, these short fragments of cDNA were PCR-amplified and electrophoresed on a 1.5–2% TAE agarose gel. From the electrophoretic agarose gel, suitable fragments (200–700 nt) were selected as templates for subsequent PCR amplification. During the QC steps, an Agilent 2100 Bioanalyzer and ABI StepOnePlus Real-Time PCR System were used in quantification and qualification of the sample library. Sequencing of the PCR-amplified library of each sample was accomplished separately in a single lane on the Illumina HiSeq™ 2000 platform (Illumina, San Diego, CA, USA) with 100-base-pair, paired-end reads.

### Raw sequence data and filtering

Sequenced data from Illumina HiSeq™ 2000 were transformed by base calling into sequence data, called the raw data or raw reads and were stored in FASTQ format. Prior to the transcriptome assembly, a stringent-filtering process of raw sequencing reads was carried out. The reads with more than 20% bases having a quality score of *Q* < 10, sequences containing more than 5% of ambiguous nucleotides or those containing adaptor sequences were removed with an in-house filter programme (Filter_fq, BGI), yielding clean data or clean reads.

### De novo transcriptome assembly

De novo ‘shot-gun’ transcriptome assembly was carried out with the short reads-assembling programme, Trinity (version release-20121005) (Grabherr et al., 2011). Three independent software modules, i.e. Inchworm, Chrysalis and Butterfly, comprised the Trinity programme and were sequentially applied to process the large volumes of RNA-seq reads. In brief, this was based on the algorithm of de Bruijn graph construction which began by aligning *k*-mers (*k* = 25), and reads with a certain length of overlap were joined to form linear contigs. The reads were mapped back onto contigs, and by referring to paired-end reads, contigs from the same transcript as well as the distances between them were determined. The contigs were then partitioned into clusters, each of which carried a complete set of de Bruijn graphs (representing the transcriptional complexity at a given gene or locus). The graphs were independently processed to obtain full-length transcripts for alternatively spliced isoforms and to tease apart transcripts that corresponded to paralogous genes.

### Transcript clustering

The transcript sequences generated through Trinity were called Unigenes. Unigenes from the transcriptome assembly were further processed for sequence splicing and redundancy removing with TGI clustering tools (TGICL) version 2.1 to acquire non-redundant (NR) transcripts at the longest possible length. The transcripts were then subjected to family clustering, which resulted in two classes of transcripts: (a) clusters, with a prefix CL and the cluster ID behind as contig; (b) singletons, whose ID was simply left with a prefix of Unigene. In each cluster, there were several transcripts with sequence similarities among them being >70%; while the singletons ‘Unigenes’ lack overlapping with other fragments at the given stringency. The value 70% was used to categorise the assembled sequences based on similarity; sequences similar to each other (may or may not be homologous as having >90% similarity) were grouped under a cluster comprising various contigs.

In the following step, the transcript Unigenes were aligned by BLASTx to protein databases in the priority order of NCBI NR, with a cut-off E < 10^−5^. Proteins with the highest ranks in the BLASTx results were referred to determine the coding region sequences of the Unigenes, followed by translation into amino acid sequences (using the standard codon table). Hence, both the nucleotide sequences (5′–3′) and amino sequences of the Unigene-coding regions were acquired. Transcript Unigenes unaligned to any of the protein databases were analysed by software named ESTScan (Iseli, Jongeneel & Bucher, 1999) to determine the nucleotide sequence (5′–3′) direction and amino sequence of the predicted coding region. The length of sequences assembled was a criterion for assembly success. To remove redundancy from each cluster, the longest sequence in each cluster was chosen as the transcript, meanwhile the length of scaffold was extended based on overlapping sequences using Phrap assembler (release 23.0) (http://www.phrap.org). The distributions of the lengths of contigs, scaffolds and Unigenes were calculated. The N50 length statistics was set at N50 > 500 for assembly success.

### Functional annotation of the transcripts

The process retrieved proteins with the highest sequence similarity with the given transcript along with their protein functional annotations, recorded in Data S1. Annotation of the transcripts provides information about the mRNA expressions (see below) and the putative protein functions. For functional annotation, the generated transcript sequences were aligned by BLASTx to protein databases NR (cut-off E < 10^−5^).

### Expression annotation of the transcripts

To determine the transcript abundances for the identified genes, the FPKM method (Mortazavi et al., 2008) was used, computed using the RNA-seq by the expectation maximisation (RSEM) tool incorporated in the assembly programme, Trinity (Li & Dewey, 2011). The formula is shown below:

$${\rm{FPKM\; of\; gene}}\;{\rm{A}} = {{{{10}^6} C} \over {N\;{\rm{L}}/{{10}^3}}},$$

where FPKM is set to be the expression of gene A, *C* to be the number of fragments (i.e. reads) that uniquely aligned to gene A, *N* to be the total number of fragments (i.e. reads) that uniquely aligned to all genes and L to be the base number in the coding sequence (CDS) of gene A. The FPKM method is able to eliminate the influence of different gene length and sequencing discrepancy on the calculation of gene expression.

### Venom gland transcript classification based on toxinology

Data S1 (from BLAST analyses) was further studied to determine which transcripts (Unigenes) could be identified as ‘toxin,’ ‘non-toxin’ and ‘unidentified’ categories. Keywords were used in search-and-find of the subject description for each toxin match. In view that the final translated toxin products are proteins in nature, the encoded amino acid sequences were subjected to a BLASTp search to ascertain homology with the latest known NCBI NR protein database restricted to the taxon Serpentes (as of 1 June 2016). Minute expression of highly similar/conserved sequences exclusive to Viperidae (vipers and pit vipers) detected by the sensitive assay were excluded from the current study for possibility of trace contamination. Transcripts for cellular proteins and house-keeping genes were categorised into ‘non-toxins’ while those without significant hits/matches were classified as ‘unidentified.’ The relative expression (FPKM) of BLAST-annotated venom gland transcriptomic Unigenes (percentage of the three categories), the relative abundance and the diversity of various toxins in percentage of (i) total protein-encoding transcripts and (ii) total toxin-encoding transcripts were determined.

### Redundancy of gene families

In addition, the redundancy of genes was assessed by dividing the transcriptional activity level or transcript reads (FPKM) with the total number of transcripts within a cluster or a group of genes. High redundancy indicates high expression level of a gene group.

### Sequence alignments

The amino acid sequences used for sequence comparison/alignments with the sequences obtained in this study were retrieved from the UniProtKB database (http://www.uniprot.org/). Multiple sequence alignment was performed with MUSCLE program (Edgar, 2004) using Jalview software v2.9 (Waterhouse et al., 2009). Pairwise sequence alignment was performed on the full-length co-expressed toxin-encoding transcripts between the two specimens with Mutalin® software (Corpet, 1988). Together with the annotated sequences, sequence comparisons were carried out and compiled in Data S3.

### Codon alignment and determination of substitution rates

The toxins nucleotide CDSs were retrieved from the nucleotide assembly file and aligned (Data S4). The non-synonymous (*K*_a_) and synonymous (*K*_s_) substitution rates per site (*K*_a_/*K*_s_) of the co-expressed transcripts were calculated using the ‘KaKs_Calculator 2.0’ (Wang et al., 2009a, 2009b, 2010). This programme implements several candidate models of codon substitution in a maximum likelihood framework. We used the approximate method, MYN method (a modified version of the Yang–Nielsen method) to estimate *K*_a_ and *K*_s_ value with default parameters. The findings were tabulated in a table available in the file of Data S4.

### Availability of supporting data

Sequence data from the venom gland transcriptome of the two NK-M and NK-T have been deposited in National Centre for Biotechnology Information (NCBI) Sequence Read Achieve (http://www.ncbi.nlm.nih.gov/Traces/sra/sra.cgi) under Bioproject: PRJNA302200 (AC ID: SRP066203) (NK-M: SRR2917658; NK-T: SRR2917657) (http://www.ncbi.nlm.nih.gov/sra/SRP066203).

## Results and Discussion

### Transcriptome assembly

**Figure 1:**
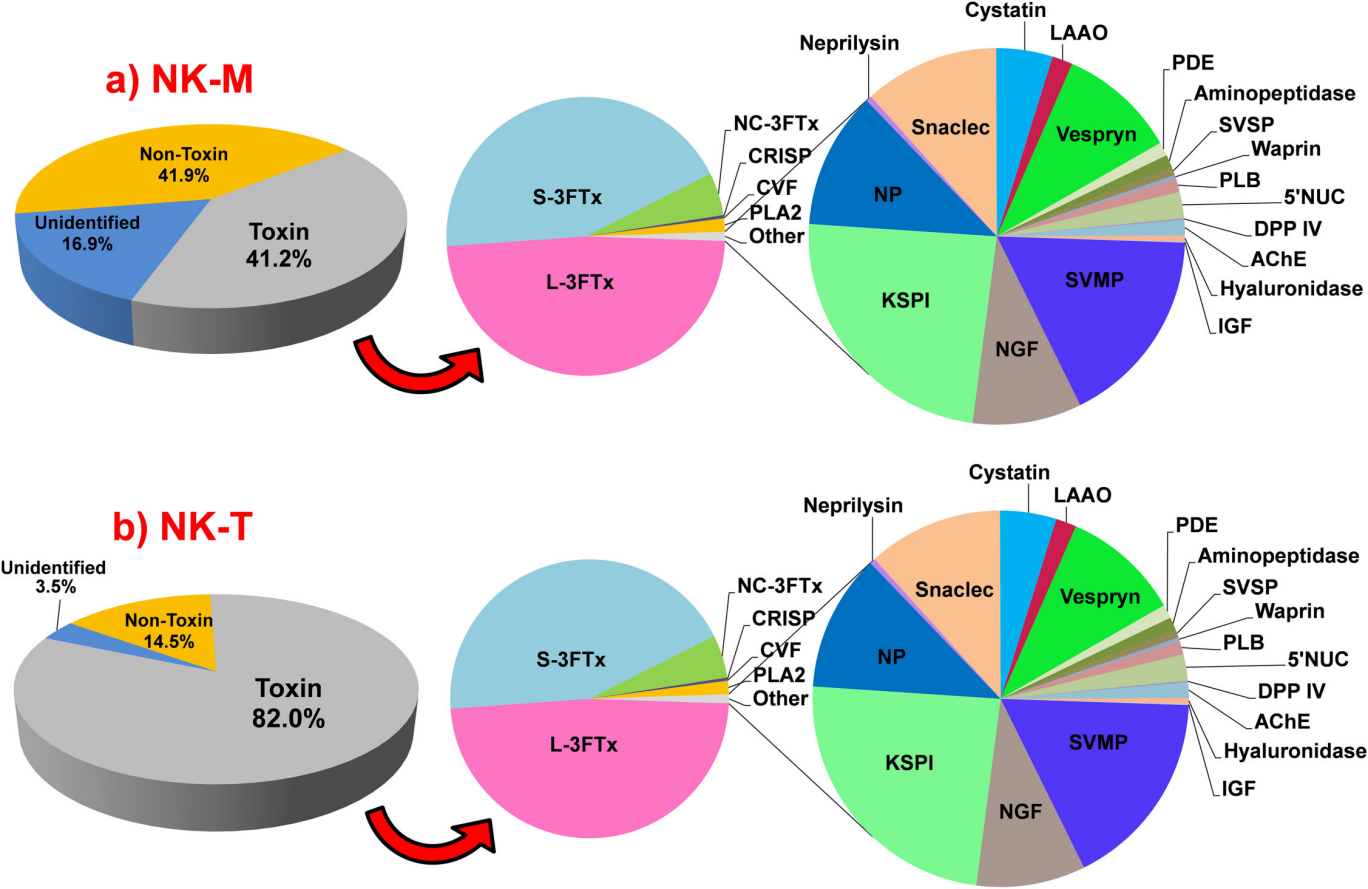
Abundance of transcripts expressed (FPKM, %) in the venom glands of *Naja kaouthia* from (a) Malaysia, NK-M; (b) Thailand, NK-T. Toxins transcripts accounted for 41.2% (NK-M) and 82.0% (NK-T) of the total FPKM, respectively. Three-finger toxin (3FTx) is the most abundant toxin family expressed in the two venom glands (NK-M, 86.8%; NK-T, 97.0% of toxin FPKM). Abbreviations: 3FTx, three-finger toxin; L, long-chain; S, short-chain; NC, non-conventional; CRISP, cysteine-rich secretory protein; CVF, cobra venom factor; PLA_2_, phospholipase A_2_; SVMP, snake venom metalloproteinase; NGF, nerve growth factor; KSPI, Kunitz-type serine proteinase inhibitor; NP, natriuretic peptide; snaclec, C-type lectin/lectin-like protein; LAAO, l-amino acid oxidase; PDE, phosphodiesterase; AP, aminopeptidase; SVSP, snake venom serine protease; PLB, phospholipase-B; 5′NUC, 5′nucleotidase; DPP-IV, dipeptidylpeptidase-IV; CFs, coagulation factor; AChE, acetylcholinesterase and IGF, insulin-like growth factor.

Illumina HiSeq 2000 sequencing was employed to sequence the transcriptome of the venom gland of two NK-M and NK-T and the data statistics were summarised as in Table 1. In NK-M, Trinity (Grabherr et al., 2011) created 145,538 contigs (N50 = 588), connected to form 73,451 Unigenes (N50 = 1,139); while in NK-T, 125,435 contigs (N50 = 547) were created and connected to form 69,840 Unigenes (N50 = 876). BLASTx alignment (E < 10^−5^) between the Unigenes and sequences in the NCBI NR protein database yielded 32,137 and 33,004 annotated Unigenes for NK-M and NK-T, respectively (Data S1). After filtering low-frequency transcripts (defined as below 10 FPKM), the assemblies were reduced and categorised into ‘unidentified,’ ‘non-toxin’ and ‘toxin’ groups (Table 1), with the ‘toxin’ group entailed the classical-venom-component proteins with neurotoxic/haemotoxic/cytotoxic properties as well as putative toxins hitherto described (Aird et al., 2013; McGivern et al., 2014; Tan et al., 2015a). Although the ‘toxin’ group, accounted for only 64 and 66 transcripts (out of more than 60,000 transcripts) in the venom gland transcriptomes of NK-M and NK-T, respectively, the expressions of these toxin genes were distinctly high, charting 41.2% (NK-M) and 82.0% (NK-T) of the total expression (FPKM). In a few elapid snakes, toxin gene expression levels have been shown to be in the range of 30–80% of total transcription (for instance, 35.3% in *Ophiophagus hannah* (Tan et al., 2015a); 45.8% in *Micrurus fulvius* (Margres et al., 2013); 70% in *Naja atra* (Jiang et al., 2011)). It appears that the rate of the overall toxin gene transcription may vary from snake to snake (perhaps affected by the condition of the snake and the time of tissue sampling), however, the relative expression of the different toxin genes is stable and can be studied compatibly between the snakes. Meanwhile, the ‘unidentified’ and ‘non-toxin’ transcripts in both transcriptomes exhibited much lower expression levels in spite of their very large number of genes (Fig. 1). The toxin genes were expressed at a high redundancy in the venom glands of both NK-M (6311.78 FPKM/transcript) and NK-T (22901.34 FPKM/transcript), respectively, compared to the non-toxin transcripts (68.18 FPKM/transcript in NK-M and 95.06 FPKM/transcript in NK-T), supporting the finding of extremely high expression of venom genes within a restricted set of families (Data S1). Overall, approximately 58.8% and 18.0% of the total FPKM in NK-M and NK-T, respectively, are unrelated to envenomation. Most of these are housekeeping genes associated with cellular metabolisms and hence transcribed at lower FPKM levels.

**Table 1:** Overview of the output statistics. The sequencing and the assembly quality of the venom gland transcriptomes of Malaysian (NK-M) and Thai Naja kaouthia (NK-T).

	NK-M	NK-T
Total raw reads	56,859,800	59,932,334
Total clean reads	53,663,062	55,186,018
Total clean nucleotides (nt)	4,829,675,580	4,966,741,620
Q20 percentage	98.28	98.31
*N* percentage	0.00	0.00
GC percentage	45.84	45.75
*Contigs created*	145,538	125,435
Total length (nt)	50,843,599	40,514,499
Mean length (nt)	349	323
N50	588	547
*Unigenes/transcripts assembled*	73,451	69,840
Total length (nt)	51,961,752	39,063,742
Mean length (nt)	707	559
N50	1139	876
*Unigenes/transcripts assembled (FPKM > 10)*	11,819	4,461
Unidentified	5,725	1,576
Non-toxin	6,030	2,819
Toxin	64	66

### Complexity of the toxin transcripts

The identified toxin transcripts from the venom glands of *N. kaouthia* from Malaysia (NK-M, 64 partial and complete transcripts, 41.2% of the total FPKM) and Thailand (NK-T, 66 partial and complete transcripts, 82.0% of the total FPKM) comprise 24 gene families, with 23 families identified for each specimen while 22 of them were co-expressed in both (Fig. 1). The fact that the 22 gene families were co-expressed in both NK-M and NK-T venom glands indicates that the toxin gene pool of NK-M and NK-T is largely conserved. Of these, 15 protein gene families were newly detected at the transcriptional level of *N. kaouthia* by the present work; these include Kunitz-type serine protease inhibitor (KSPI), natriuretic peptide (NP), neprilysin, snake venom C-type lectin/lectin-like protein (snaclec), phosphodiesterase (PDE), aminopeptidase (AP), snake venom serine protease (SVSP), waprin, phospholipase B (PLB), 5′nucleotidase (5′NUC), dipeptidylpeptidase-IV (DPP-IV), coagulation factor (CF), acetylcholinesterase (AChE), hyaluronidase (HYA) and insulin-like growth factor (IGF). Some of these toxins were detected in the recent global profiling of *N. kaouthia* venom (Laustsen et al., 2015; Tan et al., 2015d), and is in agreement with the previous enzymatic study that has clearly detected PDE, protease, 5′NUC, AChE and HYA activities in *N. kaouthia* venom (Tan & Tan, 1988).

A total of 63 and 62 NR toxin transcripts were derived from NK-M and NK-T venom glands, respectively (Table 2). Of these, 28 (NK-M) and 27 (NK-T) encoded full-length protein-encoding transcripts (defined here as >90% coverage to the protein-encoding region of the annotated protein sequences) (Tables 3 and 4). These encompass most of the medically relevant toxins of cobra venoms, including neurotoxins (LNTXs, long neurotoxins; SNTXs, short neurotoxins), cytotoxins (CTXs) and phospholipases A_2_ (PLA_2_s) of various isoforms; hence, the species-specific database established herein represents a valuable depot of bioinformation for further structural and functional studies. It was noted that sequencing of full-length protein-encoding transcripts of several larger venom components was hardly achieved in the current study, probably because Trinity, although a top-performing assembler good at estimating transcript isoforms, has limitations in capturing complete transcript sequences (Honaas et al., 2016). To obtain complete sequences of all genes, we suggest that the current assembly algorithm can be revised with possibly the incorporation of newer programming software in the future. On the other hand, it is also noted that the application of BLASTx search against NR database yielded limited matches to *N. kaouthia*-specific sequences due to the small database available for this species (Data S2A and S2B; Table 5). As outlined in the methodology, the annotation for most of the transcripts was based on sequence homology to toxins of closely related taxa available in the current repository. This first de novo transcriptomic study for *N. kaouthia* has successfully uncovered numerous novel toxin sequences and the data is expected to contribute significantly to enriching the species database (Data S3).

**Table 2:** Overview of the numbers of toxin transcripts, full-length sequences and co-expressed toxin genes from the venom gland transcriptomes of Malaysian (NK-M) and Thai *Naja kaouthia* (NK-T).

	NK-M	NK-T
1. Toxin transcripts	64	66
2. Non-redundant toxin transcripts^a^	63	62
3. Protein annotated from non-redundant toxin transcripts^b^	49	51
4. Full-length coverage transcript from non-redundant toxins transcripts^c^	28	27
5. Non-redundant toxin transcripts co-expressed in both NK-M and NK-T (according to protein accession ID)^d^		
–Total non-redundant toxins transcripts^e^	46	43
–Total protein subtypes annotated^f^	36	36

**Notes:**

a Number of toxins transcripts excluding the redundant transcripts.

b Number of annotated protein from the non-redundant toxins transcripts (one or more transcripts could be annotated to same protein ID).

c Non-redundant toxins transcripts with full-length coverage (>90%) to the protein-encoding region of the annotated protein ID.

d Non-redundant toxins transcripts co-expressed in the venom gland transcriptome of both NK-M and NK-T.

e Number of non-redundant toxins transcripts co-expressed in both NK-M and NK-T.

f Number of annotated proteins from the non-redundant toxins transcripts.

**Table 3:** List of full-length transcripts (37 in total) obtained from the venom gland transcriptomes of Malaysian *Naja kaouthia* (NK-M).

Protein family	Code	Protein ID	Annotated accession (UniProt/NCBI)	Species	A. acid (fall in mature chain)	Mature chain of accession ID	Coverage (mature chain)	Coverage (%)
3FTx	NKM_FTX01	Alpha-elapitoxin-Nk2a	P01391	*N. kaouthia*	71	71	1–71	100
	NKM_FTX05	Cobrotoxin	P60770	*N. atra*	62^a^	62	22–83	100
	NKM_FTX06	Cobrotoxin-c	P59276	*N. kaouthia*	61	61	1–61	100
	NKM_FTX07	Short neurotoxin SNTX11	Q2VBP1	*O. hannah*	56^a^	57	22–77	98.25
	NKM_FTX09	Neurotoxin homolog NL1	Q9DEQ3	*N. atra*	65^a^	65	22–86	100
	NKM_FTX10	Cytotoxin 5	Q98961	*N. atra*	60^a^	60	22–81	100
	NKM_FTX11	Cardiotoxin 7	Q91996	*N. atra*	62^a^	62	17–83	100
	NKM_FTX12	Muscarinic toxin-like protein 2	P82463	*N. kaouthia*	62	65	1–62	95.38
	NKM_FTX13	Muscarinic toxin-like protein 3 homolog	A8N286	*O. hannah*	65^a^	65	22–86	100
	NKM_FTX14	Weak tryptophan-containing neurotoxin	P82935	*N. kaouthia*	65	65	22–86	100
	NKM_FTX15	Three finger toxin-like	Q27J50	*L. muta*	73^a^	73	21–93	100
PLA_2_	NKM_PLA01	Acidic phospholipase A2 1	P00596	*N. kaouthia*	119	119	28–146	100
vNGF	NKM_NGF01	Nerve growth factor beta chain	A59218	*N. kaouthia*	116	116	131–246	100
KSPI	NKM_KPI01	Protease inhibitor	P20229	*N. naja*	57	57	1–57	100
	NKM_KPI02	Kunitz-type protease inhibitor 1	V8N7R6	*O. hannah*	488^a^	481	26–506	100^b^
Snaclec	NKM_SCL01	C-type lectin BFL-1	Q90WI8	*B. fasciatus*	137^a^	137	22–158	100
	NKM_SCL02	C-type lectin BFL-2	Q90WI7	*B. fasciatus*	137^a^	137	22–158	100
Cystatin	NKM_CYS01	Hypothetical protein L345_15265	V8NBS6	*O. hannah*	81^a^	74	1–74	100
	NKM_CYS02	Cystatin	E3P6P4	*N. kaouthia*	115	115	27–141	100^b^
LAAO	NKM_LAO01	l-amino-acid oxidase	A8QL58	*N. atra*	495^a^	430	20–449	100^b^
Vespryn	NKM_VES01	Thaicobrin	P82885	*N. kaouthia*	190	108	1–108	100^b^
PDE	NKM_PDE01	Phosphodiesterase	U3FAB3	*M. fulvius*	836^a^	836	18–853	100
SVSP	NKM_SSP01	Serine protease harobin	Q5MCS0	*H. curtus*	223^a^	232	34–265	96.12
Waprin	NKM_WAP01	Scuwaprin-a	B5G6G8	*O. scutellatus scutellatus*	51^a^	51	22–72	100
PLB	NKM_PLB01	Putative phospholipase B 81b	F8J2D3	*D. coronoides*	518^a^	518	36–553	100
DPP-IV	NKM_DPP01	Venom dipeptidylpeptidase-IV	A6MJH7	*P. australis*	753^a^	753	1–753	100
AChE	NKM_ACE01	Acetylcholinesterase	Q92035	*B. fasciatus*	551^a^	578	29–579	95.33
IGF	NKM_IGF01	Insulin-like growth factor II	V8NR69	*O. hannah*	161^a^	161	25–185	100

**Notes:**

*B*, *Bungarus*; *D*, *Drysdalia*; *H*, *Hydrophis*; *L*, *Lachesis*; *M*, *Micrurus*; *N*, *Naja*; *O*, *Ophiophagus/Oxyuranus*; *P*, *Pseudechis.*

a Novel protein newly reported for the *Naja kaouthia* species.

b Possesses sequence variance to the annotated sequence.

**Table 4:** List of full-length transcripts (44 in total) obtained from the venom gland transcriptomes of Thai *Naja kaouthia* (NK-T).

Protein family	Code	Protein ID	Annotated accession (UniProt/NCBI)	Species	A. acid (fall in mature chain)	Mature chain of Accession ID	Coverage (mature chain)	Coverage (%)
3FTx	NKT_FTX07	Short neurotoxin SNTX11	Q2VBP1	*O. hannah*	56^a^	57	22–77	98.25
	NKT_FTX08	Neurotoxin homolog NL1	Q9DEQ3	*N. atra*	65^a^	65	22–86	100
	NKT_FTX12	Cardiotoxin 7	Q91996	*N. atra*	62^a^	62	22–83	100
	NKT_FTX13	Muscarinic toxin-like protein 2	P82463	*N. kaouthia*	62	65	1–62	95.38
	NKT_FTX16	Three finger toxin-like precursor	Q27J50	*L. muta*	73^a^	73	21–93	100
CRISP	NKT_CRP02	Cysteine-rich venom protein kaouthin-2	P84808	*N. kaouthia*	207	213	26–232	97.18
PLA_2_	NKT_PLA01	Acidic phospholipase A2 1	P00596	*N. kaouthia*	119	119	28–146	100
vNGF	NKT_NGF01	Nerve growth factor beta chain precursor	A59218	*N. kaouthia*	116	116	131–246	100
KSPI	NKT_KPI01	Protease inhibitor NACI	Q5ZPJ7	*N. atra*	57^a^	57	25–81	100
	NKT_KPI02	Protease inhibitor	B2BS84	*A. labialis*	229^a^	232	21–252	98.71
	NKT_KPI03	Kunitz-type protease inhibitor 1	V8N7R6	*O. hannah*	488^a^	481	26–506	100^b^
Neprilysin	NKT_NEP01	Neprilysin-like	XP_007436944	*P. bivittatus*	672^a^	672	77–748	100
Snaclec	NKT_SCL01	C-type lectin BFL-1	Q90WI8	*B. fasciatus*	137^a^	137	22–158	100
	NKT_ SCL03	C-type lectin BFL-2	Q90WI7	*B. fasciatus*	137^a^	137	22–158	100
Cystatin	NKT_CYS01	Hypothetical protein L345_15265	V8NBS6	*O. hannah*	81^a^	74	1–74	100^b^
	NKT_CYS02	Cystatin	E3P6P4	*N. kaouthia*	115	115	27–141	100
LAAO	NKT_LAO01	l-amino-acid oxidase	A8QL58	*N. atra*	495^a^	430	20–449	100^b^
Vespryn	NKT_VES01	Thaicobrin	P82885	*N. kaouthia*	190	108	1–108	100^b^
PDE	NKT_PDE01	Phosphodiesterase	U3FAB3	*M. fulvius*	836^a^	836	18–853	100
SVSP	NKT_SSP01	Serine protease harobin	Q5MCS0	*H. curtus*	223^a^	232	34–265	96.12
Waprin	NKT_WAP01	Scuwaprin-a	B5G6G8	*O. scutellatus scutellatus*	48^a^	51	25–72	94.12
PLB	NKT_PLB01	Putative phospholipase B 81b	F8J2D3	*D. coronoides*	518^a^	518	36–553	100
5′NUC	NKT_NUC01	Snake venom 5′nucleotidase	B6EWW8	*G. brevicaudus*	524^a^	524	41–564	100
DPP-IV	NKT_DPP01	Venom dipeptidylpeptidase-IV	A6MJH7	*P. australis*	753^a^	753	1–753	100
AChE	NKT_ACE01	Acetylcholinesterase	Q92035	*B. fasciatus*	578^a^	578	29–606	100
HYA	NKT_HYA01	Hyaluronidase	A3QVN2	*E. ocellatus*	426^a^	426	24–449	100
IGF	NKT_IGF01	Insulin-like growth factor I isoform X2	XP_007420002	*P. bivittatus*	168^a^	155	1–155	100^b^

**Notes:**

*A*, *Austrelaps*; *B*, *Bungarus*; *D*, *Drysdalia*; *E*, *Echis*; *G*, *Gloydius*; *H*, *Hydrophis*; *L*, *Lachesis*; *M*, *Micrurus*; *N*, *Naja*; *O*, *Ophiophagus/Oxyuranus*; *P*, *Pseudechis/Python.*

a Novel protein newly reported for the *Naja kaouthia* species.

b Possesses sequence variance to the annotated sequence.

**Table 5:** Overview of the families and subtypes of toxin genes in the venom gland transcriptomes of Malaysian (NK-M) and Thai *Naja kaouthia* (NK-T).

Protein family	Subtype	Accession/species	NK-M^b^, % (non-redundant transcript)	NK-T^b^, % (non-redundant transcript)
Three-finger toxin (3FTx)			86.84 (15)	97.03 (16)
	LNTX		1.68 (4)	48.32 (3)
	Alpha-elapitoxin-Nk2a	P01391 (*N. kaouthia*)^a^	1.52 (1)^c^	48.32 (2)
	Hypothetical protein L345_18084	V8N212 (*O. hannah*)	–	0.00 (1)
	Long neurotoxin-like OH-31	Q53B55 (*O. hannah*)^a^	0.16 (3)	–
	SNTX		46.43 (5)	22.71 (6)
	Cobrotoxin	P60770 (*N. atra*)^a^	16.45 (1)^c^	3.53 (1)
	Cobrotoxin-c	P59276 (*N. kaouthia*)^a^	14.46 (1)^c^	18.38 (2)
	Short neurotoxin SNTX11	Q2VBP1 (*O. hannah*)^a^	12.44 (1)^c^	0.67 (1)^c^
	Cobrotoxin-b	P80958 (*N. atra*)	2.37 (1)	–
	Neurotoxin homolog NL1	Q9DEQ3 (*N. atra*)^a^	0.71 (1)^c^	0.12 (1)^c^
	Neurotoxin-like protein NTL2	Q9W717 (*N. atra*)	–	0.01 (1)
	CTX		29.01 (2)	20.54 (3)
	Cytotoxin 3	P01446 (*N. atra*)	–	18.35 (1)
	Cytotoxin 5	Q98961 (*N. atra*)^a^	27.20 (1)^c^	1.17 (1)
	Cardiotoxin 7	Q91996 (*N. atra*)^a^	1.81 (1)^c^	1.02 (1)^c^
	MTLP		0.72 (2)	0.47 (1)
	Muscarinic toxin-like protein 2	P82463 (*N. kaouthia*)^a^	0.71 (1)^c^	0.47 (1)^c^
	Muscarinic toxin-like protein 3 homolog	A8N286 (*O. hannah*)	0.01 (1)^c^	–
	WTX		9.01 (2)	4.97 (3)
	Weak tryptophan-containing neurotoxin	P82935 (*N. kaouthia*)^a^	9.00 (1)^c^	4.12 (1)
	Probable weak neurotoxin NNAM2	Q9YGI4 (*N. kaouthia*)	–	0.85 (1)
	Three finger toxin-like	Q27J50 (*L. muta*)^a^	0.01 (1)^c^	0.00 (1)^c^
Cysteine-rich secretory protein (CRISP)			4.03 (5)	0.31 (5)
	Kaouthin-2	P84808 (*N. kaouthia*)^a^	2.10 (2)	0.11 (1)^c^
	Natrin-1	Q7T1K6 (*N. atra*)^a^	1.41 (2)	0.20 (3)
	Natrin-2	Q7ZZN8 (*N. atra*)	0.52 (1)	0.01 (1)
Cobra venom factor (CVF)			2.19 (4)	0.06 (2)
	Cobra venom factor	Q91132 (*N. kaouthia*)^a^	2.19 (4)	0.06 (2)
Phospholipase A_2_ (PLA_2_)			1.80 (2)	1.54 (1)
	Acidic phospholipase A2 1	P00596 (*N. kaouthia*)^a^	1.79 (1)^c^	1.54 (1)^c^
	Phospholipase A2 GL16-1	Q8JFB2 (*L. semifasciata*)	0.01 (1)	–
Snake venom metalloproteinase (SVMP)			1.62 (6)	0.18 (10)
	Zinc metalloproteinase-disintegrin atragin	D3TTC2 (*N. atra*)	1.03 (2)	0.03 (1)
	Haemorrhagic metalloproteinase-disintegrin kaouthiagin	P82942 (*N. kaouthia*)	0.53 (3)	–
	Carinatease-1	B5KFV1 (*T. carinatus*)	0.06 (1)	0.01 (1)
	Zinc metalloproteinase-disintegrin NaMP	A8QL59 (*N. atra*)	–	0.01 (4)
	Nigrescease-1	B5KFV8 (*C. nigrescens*)	–	0.04 (1)
	Zinc metalloproteinase-disintegrin cobrin	Q9PVK7 (*N. kaouthia*)	–	0.05 (1)
	Zinc metalloproteinase-disintegrin atrase-A	D5LMJ3 (*N. atra*)	–	0.02 (1)
	Zinc metalloproteinase mocarhagin	Q10749 (*N. mossambica*)	–	0.02 (1)
Nerve growth factor (NGF)			1.04 (1)	0.10 (1)
	Nerve growth factor beta chain	A59218 (*N. kaouthia*)^a^	1.04 (1)^c^	0.10 (1)^c^
Kunitz-type serine protease inhibitor (KSPI)			0.63 (2)	0.25 (3)
	Protease inhibitor	P20229 (*N. naja*)	0.61 (1)^c^	–
	Protease inhibitor NACI	Q5ZPJ7 (*N. atra*)	–	0.24 (1)^c^
	Kunitz-type protease inhibitor 1	V8N7R6 (*O. hannah*)^a^	0.02 (1)^c^	0.00 (1)^c^
	Protease inhibitor	B2BS84 (*A. labialis*)^a^	–	0.01 (1)^c^
Natriuretic peptide (NP)			0.56 (2)	0.12 (2)
	Natriuretic peptide Na-NP	D9IX97 (*N. atra*)^a^	0.54 (2)	0.12 (2)
Neprilysin			0.37 (3)	0.01 (1)
	Neprilysin-like	XP_007436944 (*P. bivittatus*)^a^	0.37 (3)	0.01 (1)^c^
Snake venom C-type lectin/lectin-like protein (snaclec)			0.31 (4)	0.12 (2)
	C-type lectin BFL-1	Q90WI8 (*B. fasciatus*)^a^	0.29 (1)^c^	0.12 (1)^c^
	C-type lectin BFL-2	Q90WI7 (*B. fasciatus*)^a^	0.02 (1)^c^	0.00 (1)^c^
	Venom C-type lectin mannose binding isoform 2 variant 1	D2YVL4 (*C. nigrescens*)	0.00 (1)	–
	C-type lectin isoform 3	H8PG91 (*P. nigriceps*)	0.00 (1)	–
Cystatin			0.19 (4)	0.05 (5)
	Hypothetical protein L345_15265	V8NBS6 (*O. hannah*)^a^	0.14 (1)^c^	0.04 (1)^c^
	Cystatin	E3P6P4 (*N. kaouthia*)^a^	0.02 (1)^c^	0.00 (1)^c^
	Cystatin-C	V8NX38 (*O. hannah*)^a^	0.02 (1)	0.00 (1)
	Hypothetical protein L345_15526	V8NB07 (*O. hannah*)	0.00 (1)	0.00 (1)
	Hypothetical protein L345_14827	V8NCS2 (*O. hannah*)	–	0.00 (1)
l-amino-acid oxidase (LAAO)			0.12 (1)	0.02 (1)
	l-amino-acid oxidase	A8QL58 (*N. atra)*^a^	0.12 (1)^c^	0.02 (1)^c^
Vespryn			0.12 (1)	0.11 (1)
	Thaicobrin	P82885 (*N. kaouthia*)^a^	0.12 (1)^c^	0.11 (1)^c^
Phosphodiesterase (PDE)			0.11 (2)	0.01 (1)
	Phosphodiesterase	U3FAB3 (*M. fulvius*)^a^	0.11 (2)^c^	0.01 (1)^c^
Aminopeptidase			0.03 (2)	0.01 (2)
	Aminopeptidase N	B6EWW5 (*G. brevicaudus*)^a^	0.02 (1)	0.01 (2)
	Aminopeptidase B	V8N861 (*O. hannah*)	0.01 (1)	–
Snake venom serine protease (SVSP)			0.03 (1)	0.01 (1)
	Serine protease harobin	Q5MCS0 (*H. curtus*)^a^	0.03 (1)^c^	0.01 (1)^c^
Waprin			0.01 (1)	0.00 (1)
	Scuwaprin-a	B5G6G8 (*O. scutellatus scutellatus*)^a^	0.01 (1)^c^	0.00 (1)^c^
Phospholipase B (PLB)			0.01 (1)	0.01 (1)
	Putative phospholipase B 81b	F8J2D3 (*D. coronoides*)^a^	0.01 (1)^c^	0.01 (1)^c^
5′nucleotidase (5′NUC)			0.01 (2)	0.02 (1)
	5′nucleotidase	A6MFL8 (*D. vestigiata*)^a^	0.00 (1)	–
	Snake venom 5′nucleotidase	F8S0Z7 (*C. adamanteus*)	0.00 (1)	–
	Snake venom 5′nucleotidase	B6EWW8 (*G. brevicaudus*)^a^	–	0.02 (1)^c^
Dipeptidylpeptidase-IV (DPP-IV)			0.01 (1)	0.00 (1)
	Venom dipeptidylpeptidase-IV	A6MJH7 (*P. australis*)^a^	0.01 (1)^c^	0.00 (1)^c^
Coagulation factor (CF)			0.01 (1)	0.00 (0)
	Coagulation factor X isoform 1	V8PHG1 (*O. hannah*)	0.01 (1)	–
Acetylcholinesterase (AChE)			0.00 (1)	0.01 (1)
	Acetylcholinesterase	Q92035 (*B. fasciatus*)^a^	0.00 (1)^c^	0.01 (1)^c^
Hyaluronidase			0.00 (0)	0.01 (2)
	Hyaluronidase	A3QVN2 (*E. ocellatus*)^a^	–	0.01 (2)^c^
Insulin-like growth factor (IGF)			0.01 (1)	0.00 (1)
	Insulin-like growth factor II	V8NR69 (*O. hannah*)^a^	0.00 (1)^c^	–
	Insulin-like growth factor I isoform X2	XP_007420002 (*P. bivittatus*)	–	0.00 (1)^c^

**Notes:**

The number in bracket represents the number of non-redundant transcript.

*A*, *Austrelaps*; *B*, *Bungarus*; *C*, *Crotalus/Cryptophis*; *D*, *Demansia/Drysdalia*; *E*, *Echis*; *G*, *Gloydius*; *H*, *Hydrophis*; *L*, *Lachesis/Laticauda*; *M*, *Micrurus*; *N*, *Naja*; *O*, *Ophiophagus/Oxyuranus*; *P*, *Parasuta/Pseudechis/Python*; *T*, *Tropidechis.*

a Toxin transcripts used in the sequence comparative study of NK-M and NK-T (Data S3 and S4).

b Level of expression in percentage (%) by FPKM (fragments per kilobase of exon model per million mapped reads).

c Transcript with full-length protein-encoding region coverage (>90%) to the mature chain of annotated protein ID.

The transcriptomes of both NK-M and NK-T venom glands were extremely biased towards three-finger toxin (3FTxs) expressions (86.8%, NK-M; 97.0%, NK-T, in total FPKM of toxins). Similarly high expression levels of 3FTX genes above 80% to >90% of total toxin expression were reported previously from the venom gland transcriptomes of the Chinese cobra (*N. atra*, 95.8%) and Malaysian king cobra (*O. hannah*, 84.9%) (Jiang et al., 2011; Tan et al., 2015a). This is consistent with the dominance of 3FTx proteins in *N. kaouthia* venom from both localities and these toxins constitute the key venom principles that are important biologically (in predation) and medically (in envenomation) (Tan et al., 2015d). In NK-M, other toxins transcripts include coding cysteine-rich secretory proteins (CRISPs, 4.03%), cobra venom factor (CVF, 2.19%), PLA_2_s (1.80%), snake-venom metalloproteinases (SVMPs, 1.62%), nerve growth factors (NGFs, 1.04%) and 17 protein families that were expressed at very low abundance (<1%): KSPIs, NPs, neprilysin, snacles, cystatins, l-amino acid oxidases (LAAOs), vespryn, PDEs, APs, SVSPs, waprins, PLBs, 5′NUCs, DPP-IV, CFs, AChE and IGFs (Fig. 1). Similarly, the expression of other toxin genes in NK-T venom gland is relatively suppressed: while PLA_2_s transcripts charted 1.54% FPKM, and those of the other 21 families are all expressed at a level below 1% FPKM. Table 5 shows the comparative gene expression profile of NK-M and NK-T venom glands. The sequences and parametric details of the transcripts were sorted according to gene families and compiled in Data S2A and S2B.

### Co-expression of toxin genes between NK-M and NK-T

Table 2 shows the key comparison of the venom gland transcriptomes between NK-M and NK-T (also see Data S2A, S2B, S3 and Table 5 for details). Among the NR toxin transcripts, 46 and 43 transcripts from NK-M and NK-T venom glands, respectively, were found to encode the same 36 toxins (Table 2). Of these, selected transcripts with at least 50% coverage of protein-encoding region were compared for sequence similarity (NK-M, 40 transcripts; NK-T, 36 transcripts) (Data S3). In total, 33 pairs of transcripts (including redundancy of allelic variation) encoding 32 different toxin genes were analysed, which encoded 18 of the 24 protein families of toxins (Data S3). The results revealed that most of the transcripts encoding the same toxin are highly conserved between NK-M and NK-T, with only minor divergence at their amino acid sequences (Data S3). Out of the 33 pairs of sequence comparison (at amino acid level), 29 pairs were either identical or with <2% divergence; two transcripts varied by 2–10%. Only two transcripts are found to exhibit >10% variation in their amino acid sequences. In theory, the three-dimensional structure of protein is expected to change markedly beyond the ‘twilight zone’ when the amino acid sequence deviates by >30% (Bordoli et al., 2009; Khor et al., 2015). The findings are indicative of a high degree of conserved CDSs in the toxin genes of NK-M and NK-T, and this is supported by results from nucleotide substitution analyses (Data S4), where the nucleotide CDSs of the co-expressed genes were analysed using simple substitution rates analyses program, KaKs_Calculator 2.0. Of note, by analysing the nine pairs of toxins which showed variable sequences, there are no significant findings on positive selection as the mean for genetic differences between the two geographical cobras. The observed sequence conservation in the genes hence may be a result of purifying selection, and the genes are constrained to engage in highly specific interactions with the conserved proteins. Nonetheless, the expression patterns of the conserved genes vary remarkably between NK-M and NK-T, implying complex regulatory processes that the result in the up-regulation of gene-encoding certain toxins, or alternatively, enhanced transcript degradation or lack of transcription that reflects pseudogenisation of certain traits. Variation in the final gene products, i.e. venoms between NK-M and NK-T is indeed conspicuous, where key α-neurotoxins differ remarkably in their relative protein abundance between the two geographical venoms (Tan et al., 2015d). In the present study, the large differences in the principal toxin expression between NK-M and NK-T were most likely the result of directional selection, potentially due to local selective pressures and/or coevolution with distinct prey populations or species. Adaptive expression variations have been well documented in animals including venomous snakes, where the expression variability has been shown to account for the differences in venom function, particularly intraspecific venom function (Lamichhaney et al., 2015, 2016; Margres et al., 2016; Zhang & Reed, 2016).

The clustering of other expressed genes as ‘toxins’ in this study generally follows the conventional ‘toxin’ classification from previous reports (Aird et al., 2013; Jiang et al., 2011; Margres et al., 2013; Rokyta et al., 2012). However, it is worthwhile to note that the expression of those ‘lesser’ gene families in the venom gland could well be by physiological default to some extent, as demonstrated by the expression of venom gene homologues across different tissues of non-venomous snakes (Hargreaves et al., 2014; Reyes-Velasco et al., 2015), and the equivocal functionality of these ‘toxins.’ Presumably, the genes were present along the continuum of a toxin-recruitment model, but have been maintaining at a nearly neutral, intermediate landscape, that is likely a result of purifying selection. For instance, SVMP and SVSP, typical viperid haemotoxins, are still expressed but at low levels in the venom glands of NK-M and NK-T. This phenomenon of relatively lower expression of SVSP and SVMP in elapids (<5%, in comparison to the levels of 20–30% in the viperid-venom gland transcriptomes) has also been shown in several other elapid-venom gland transcriptomes (Casewell et al., 2009; Correa-Netto et al., 2011; Jiang et al., 2011; Junqueira-de-Azevedo et al., 2015; Margres et al., 2013; Tan et al., 2015a).

### Correlation between transcriptome and proteome

Of the 24 toxin gene families expressed in *N. kaouthia* venom gland, only 13 were reported at the protein level as shown in a recent comparative proteomic study (detectable proteins being 3FTx, PLA_2_, CRISP, SVMP, LAAO, CVF, KSPI, NP, PDE, 5′NUC, vespryn, snaclec and NGF) (Tan et al., 2015d). This shows that a relatively large number of putative toxins were never or only translated and secreted into the venom gland in a minute amount. Several studies have shown that there is no positive correlation between levels of venom-gene expression and protein abundance (Durban et al., 2011; Tan et al., 2015a); although some other studies showed conflicting results (Aird et al., 2013; Casewell et al., 2014; Rokyta, Margres & Calvin, 2015), presumably because of the complex regulation processes governing the mRNA half-life, translation and protein maturation (Vogel & Marcotte, 2012). Admittedly, the issue of correlation between transcriptome and proteome is a complex one (Li, Bickel & Biggin, 2014), as the analysis of the individual gene expression could be further affected by practical factors, e.g. the time span between venom collection and gland tissue harvesting. In this study, the venom glands were sampled four days after venom milking as a way to maximise the yield of total mRNA (Rotenberg, Bamberger & Kochva, 1971), although the different genes might be expressed at different rates.

### Three-finger toxins

**Figure 2:**
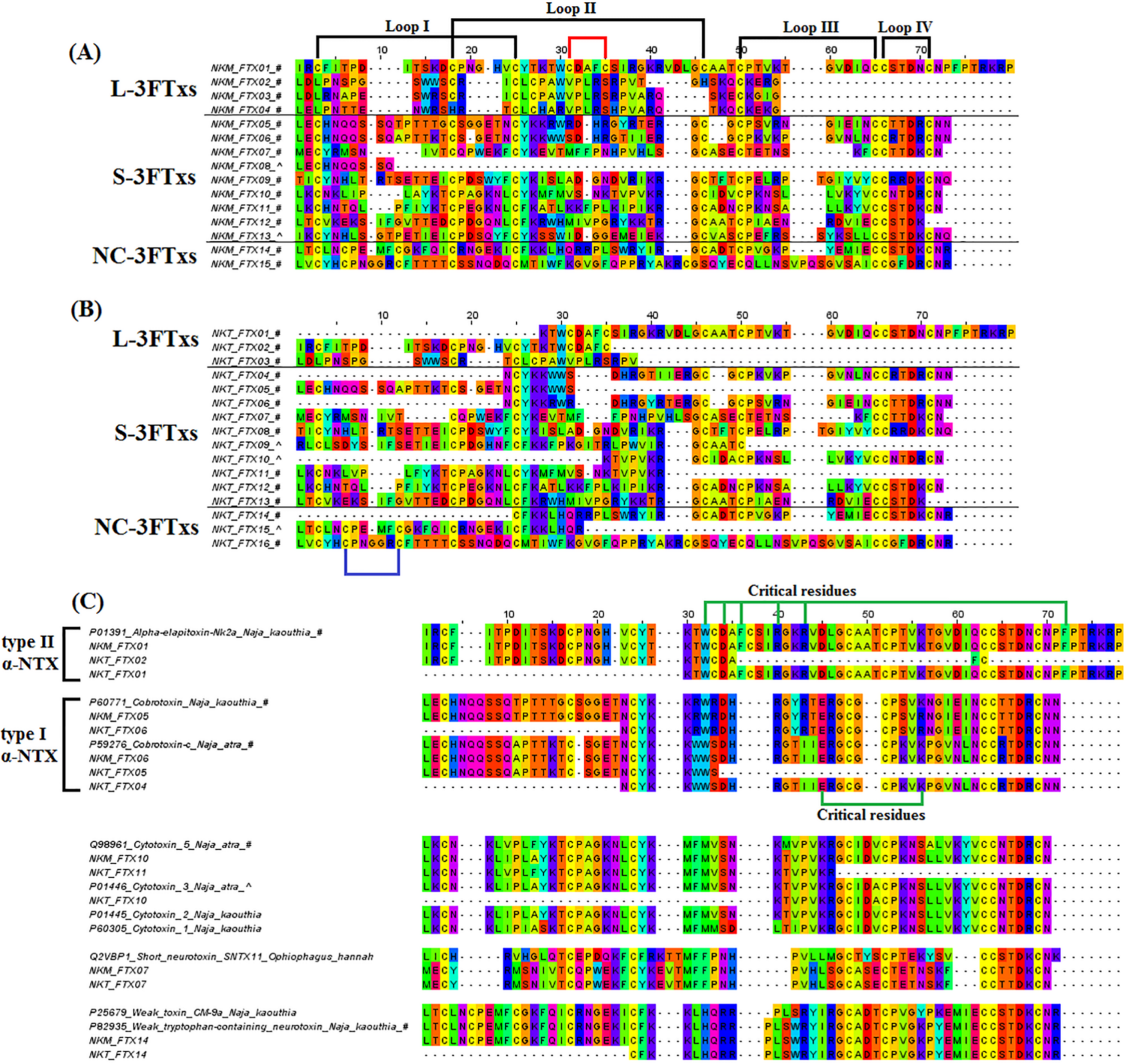
Multiple sequence alignment of three-finger toxin (3FTx) transcripts from the venom gland transcriptomes of NK-M and NK-T. (A) Malaysian *N. kaouthia* 3FTxs were aligned to show disulphide bonding. (B) Thai *N. kaouthia* 3FTxs were aligned to show disulphide bonding. (C) 3FTxs were aligned and compared to sequences from the annotated public database sequence. Black, disulphide bond loops; blue, additional disulphide bond of S-3FTxs; red, additional disulphide bond of L-3FTxs; green, critical residues of S-3FTx; #, toxins co-expressed in the NK-M and NK-T; ^, toxin transcripts expressed in either source.

Three-finger toxins typically constitute the main bulk of toxins in elapid venoms, in particular venoms of the cobras, king cobra and some sea snakes (Tan et al., 2015b; Tan & Tan, 2015). The mini-proteins fold in a similar pattern, with three β-stranded loops extending from a central core containing four to five conserved disulphide bridges (Hedge et al., 2009). In spite of the structural similarity, 3FTxs exhibit a wide range of pharmacological activities and they are conventionally classified further into different subtypes (Chanda et al., 2013; Kini & Doley, 2010). The orthologues of 3FTx genes may have been existent in the ancestral state prior to the divergence of different caenophidian lineage and venom-gene expansion (Reyes-Velasco et al., 2015), but adaptation and differential expression pattern follow in the different lineages over long evolutionary time. While 3FTx genes are highly expressed (>80%) in most elapids such as king cobra (Tan et al., 2015a; Vonk et al., 2013) and the monocled cobra as shown here, some studies have reported the transcription of 3FTx genes (albeit at very low levels) in the venom gland of some viperids and colubrids (Aird et al., 2013; Pawlak et al., 2006; Rokyta et al., 2012), or even in the rictal gland of python (Reyes-Velasco et al., 2015), but generally there is lesser isoform variability in these species. Our transcriptomic results showed that 3FTxs were highly and diversely expressed in both *N. kaouthia* venom glands, with a total of 15 and 16 transcripts identified in NK-M and NK-T, respectively. These transcripts were further classified into long-chain (L-3FTx, represented by LNTX: NK-M, four transcripts; NK-T, three transcripts), short-chain (S-3FTx, including SNTX, CTX and muscarinic toxin-like proteins (MLTPs): NK-M, nine transcripts; NK-T, 10 transcripts) and non-conventional (NC) 3FTxs (NC-3FTx, including weak toxins (WTXs): NK-M, two transcripts; NK-T, three transcripts), according to the classification system based on the number and position of disulphide bonds (Kini & Doley, 2010) (Data S2A and S2B; Fig. 2).

Altogether, a total of 11 and five full-length 3FTx transcripts were uncovered for NK-M and NK-T, respectively (Data S2A and S2B). Among these 3FTx transcripts, seven and four novel sequences were identified for NK-M and NK-T, respectively (Tables 3 and 4). The CDSs of the 11 3FTx genes that were found co-expressed in both NK-M and NK-T were largely conserved, implying that these paralogous sequences have been maintained by evolution despite potential allopatric subspeciation (Data S4). Although the genetic divergence is lacking within the 3FTxs, the expression levels of α-neurotoxin genes are noted to vary substantially between NK-M and NK-T (Fig. 1; Table 5). In the NK-M venom gland, S-3FTxs are the dominantly expressed transcripts, whereas in the NK-T venom gland, L-3FTxs are much more abundantly expressed (Table 5). The findings suggest that the transcription of certain toxin genes may have been selectively suppressed or the up-regulation in other toxin transcripts. A more specific example is the expression of the LNTX, α-elapitoxin-Nk2a (UniProtKB: P01391), a well-investigated long neurotoxin from Thai *N. kaouthia* venom. This unique LNTX gene was found to be co-expressed in both NK-M and NK-T (NKM_FTX01, NKT_FTX01 and NKT_FTX02) but vary significantly between the two at the expression level. While α-elapitoxin-Nk2a accounts for 48.3% of toxin gene expression in the NK-T venom gland, its expression is extremely low in the NK-M venom gland, contributing a mere 1.7% of total toxin transcripts. The marked difference in the expression level of this toxin in the respective venom gland of NK-T and NK-M is consistent with its abundance in the venom proteome of NK-T and NK-M (33.3% and 3.9% of total venom protein, respectively) (Tan et al., 2015d). On the other hand, SNTXs are the most abundantly expressed among the transcripts of short-chain 3FTxs (22.68% and 46.39% of toxin gene expression in NK-T and NK-M, respectively), followed by CTXs/cardiotoxins (CTXs, 20.52% and 28.99% in NK-T and NK-M, respectively), whereas MLTPs are expressed at a much lower level in both (MTLPs, 0.47% and 0.71%, respectively in NK-T and NK-M) (Table 5). In contrast to LNTX, SNTXs were expressed at a relatively higher level in NK-M (46.39%) than in NK-T (22.68%). This distinct expression of SNTX was, however, not reflected in the minor difference in SNTX content of the two venoms (4.2% and 7.7% of total venom proteins in NK-M and NK-T, respectively) (Tan et al., 2015d).

The SNTX transcripts NKM_FTX05 and NKT_FTX06 show amino acid sequence identical to cobrotoxin from *N. atra* (UniProtKB: P60770), whereas NKM_FTX06, NKT_FTX04 and NKT_FTX05 have amino acid sequences identical to cobrotoxin-c (UniProtKB: P59276) from *N. kaouthia* (Meng et al., 2002; Yang, Yang & Huang, 1969). It is interesting to note that all three cobrotoxins annotated (the former two transcripts, and NKM_FTX08 that is homologous to cobrotoxin-b; UniProtKB: P80958) have been previously reported in the venom of *N. kaouthia* from Yunnan, China (Meng et al., 2002; Qiumin et al., 2002), indicating that the cobrotoxin-encoding gene is well conserved in *N. kaouthia* from distant geographical regions. These SNTXs are highly lethal α-neurotoxins in rodents although some studies suggested that cobra venom SNTXs exhibit a weaker affinity towards the nicotinic acetylcholine receptor (nAChR) as compared to LNTXs, due to the lack of the fifth disulphide bridge in its structure (Barber, Isbister & Hodgson, 2013; Servent et al., 1997, 2000). The weaker affinity of SNTX towards nAChR, however, does not compromise its lethal potency (having compatible LD_50_ with LNTX), but has important practical concern as its poor antigenicity is a contributing factor to low neutralisation efficacy of antivenom against SNTX-predominated venoms (Leong et al., 2015; Tan et al., 2015b, 2016a).

Structurally resembling the SNTXs, CTXs are basic proteins with hydrophobic three-finger loops that can interact with the phospholipid bilayer of cell membranes, thereby mediating cytotoxic effect (Feofanov et al., 2005; Konshina et al., 2011; Osipov et al., 2008; Tan & Tan, 2016). CTX genes are highly expressed in *N. kaouthia* venom glands, with transcripts constituting 28.99% and 20.52% of the total toxin FPKM in NK-M and NK-T, respectively. Overall, transcripts highly homologous to three different CTX genes were detected: CTX-3 (UniProtKB: P01446), CTX-5 (UniProtKB: Q98961) and CTX-7 (UniProtKB: Q91996). Transcript NKT_FTX10, coding CTX-3, was solely expressed in NK-T transcriptome at a significant level (18.35%), representing a potential unique biological marker from the venom gland transcriptome of NK-T. Besides, NKM_FTX10 and NKT_FTX11, expressed respectively by NK-M and NK-T, are homologous to CTX-5 but sequence variation is noted between the two geographical variants. Meanwhile, NKM_FTX11 and NKT_FTX12, co-expressed in both NK-M and NK-M venom glands, exhibit identical sequences to CTX-7 (reported from *N. atra*), indicating that this CTX gene is relatively well-conserved within *N. kaouthia* from Thailand and Malaysia as well as the Taiwanese *N. atra* (Data S3; Table 5; Fig. 2C).

The current transcriptomic study also reveals the expression of MTLPs and WTXs, two subgroups of venom neurotoxins, which have not been extensively investigated. MTLPs were reported to have low affinity towards muscle and neuronal-type receptors (nAChRs) (Kukhtina et al., 2000), while WTXs are weak antagonist to cholinergic receptors and non-lethal to mice by intravenous route, up to 10 mg/kg (Utkin et al., 2001a, 2001b). MTLP is the least expressed 3FTxs in the venom glands of both NK-M (two transcripts, 0.71%) and NK-T (one transcript, 0.47%) (Data S3; Table 5). In contrast, the expression levels of WTXs are relatively higher (NK-M, 9.01%; NK-T, 4.97%), with weak tryptophan-containing neurotoxin (UniProtKB: P82935) being the most abundant within this subgroup. The weak neurotoxin was suggested to dose-dependently suppress the orientation–exploration and locomotion activities, as well as to cause weak neurotropic effects in rodents, possibly involving both nicotinic and muscarinic acetylcholine receptors (Mordvintsev et al., 2007). However, its relevance to the pathogenesis of cobra envenomation in human remains to be further elucidated.

### Phospholipase A_2_

**Figure 3:**
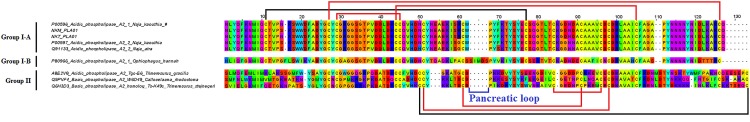
Multiple sequence alignment of phospholipase A_2_ (PLA_2_) transcripts from the venom gland transcriptomes of NK-M and NK-T in comparison to PLA2 sequences of representative venomous snakes. Red, conservative disulphide bonds; black, additional disulphide bond; blue, residues of pancreatic loops; #, toxins co-expressed in the NK-M and NK-T; ^, toxin transcripts expressed in either source.

A total of two and one PLA_2_ transcripts were identified from NK-M and NK-T venom glands respectively. Among these, NKM_PLA01 and NKT_PLA01 are the predominantly expressed forms of PLA_2_ and they are homologous to Group-IA acidic PLA_2_-1 (UniProtKB: P00596) isolated from the venom of *N. kaouthia* from Thailand (Joubert & Taljaard, 1980) (Fig. 3). From the sequence obtained, NKT_PLA01 was identical to the previously reported sequence (UniProtKB: P00596), while NKM_PLA01 is highly homologous with only one amino acid differing between the two, at the non-functional-critical site (Data S3; Fig. 3). Snake venom PLA_2_ commonly exists in multiple isoforms and exhibits great diversity in biological properties (Kini, 2003), however, it has not been easy to pinpoint the residues or segments of PLA_2_ that govern the pharmacological effects (Doley, Zhou & Kini, 2009). In agreement with the proteomic study and purified toxin characterisation (Tan et al., 2015d, 2016a), the predominant form of PLA_2_ in *N. kaouthia* venom is the acidic, enzymatic PLA_2_, shown with conserved Asp-49 residue in the current study. The acidic Asp-49-PLA_2_, however, was not lethal in mice even at a dose of >10–20 times of the median lethal dose of whole venom (Tan et al., 2016a). This is consistent with the report of non-toxic property of various acidic-type PLA_2_s isolated from the venoms of Indian *N. kaouthia* (Joubert & Taljaard, 1980) and Pakistani *N. naja* (Wong, Tan & Tan, 2016), but diverged markedly from the highly lethal, neutral/basic venom PLA_2_s characterised for other Southeast Asian cobras including *Naja sumatrana* and *N. sputatrix* (Leong et al., 2015; Tan & Arunmozhiarasi, 1989). The acidic PLA_2_s hence may serve a secondary role of ancillary function, for instance, potentiating the toxic actions of other venom components, including CTXs/cardiotoxins (Gasanov, Dagda & Rael, 2014), SVMPs (Bustillo et al., 2015) and weak neurotoxin (Mukherjee, 2010) to enhance tissue damages which are crucial for prey digestion but clinically deleterious as it complicates local tissue necrosis. On the other hand, although the mRNA level of the predominant form of PLA_2_ was relatively low (∼2%) within the venom gland, the PLA_2_ protein content in *N. kaouthia* venom is disproportionately higher (12–14% of total venom proteins) (Laustsen et al., 2015; Tan et al., 2015d). A possible explanation for this is that the mRNA of the PLA_2_ may have longer half-life, and thus a low mRNA level is sufficient to produce the necessary PLA_2_ protein (Vogel & Marcotte, 2012).

### Cysteine-rich secretory protein

**Figure 4:**
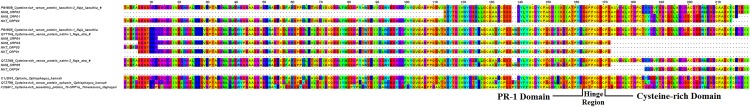
Multiple sequence alignment of cysteine-rich secretory protein (CRISP) transcripts from the venom gland transcriptomes of NK-M and NK-T in comparison to CRISP sequences of other venomous snake. Black, differentiate PR-1 domain/hinge region/cysteine-rich domain; #, toxins co-expressed in the NK-M and NK-T; ^, toxin transcripts expressed in either source.

Although the role of CRISP in the pathogenesis of snake envenomation remains unclear, its occurrence in a wide range of snake venoms indicates that it may play a certain role in the predator envenomation strategies (Sunagar et al., 2015). In view of the protein’s limited diversity and little evidence of gene duplication, it has been suggested that the CRISP gene family is functionally conserved across most lineages (Vonk et al., 2013). The current study showed that five CRISP transcripts obtained from each of the NK-M and NK-T venom gland were all highly homologous to sequences of known snake venom CRISPs (Fig. 4). Of these, the major CRISP transcript in NK-M venom gland was annotated to kaouthin-2 (UniProtKB: P84808), a protein that has been isolated from *N. kaouthia* venom (unspecified geographical origin) (Osipov et al., 2005), whereas natrin-1 (UniProtKB: Q7T1K6) (Jin et al., 2003), a CRISP isolated from *N. atra* venom (Kunming, China) was highly expressed in the NK-T venom gland (Data S2A and S2B; Table 5). Even though there are substantial variations in the amino acid sequences between kaouthin and natrin, the two CRISPs were reported to exhibit similar pharmacological activities such as antagonizing the calcium-activated (KCa) channel, voltage-gated potassium channel (Kv) and calcium release channel/ryanodine receptor (RyR) (Chang et al., 2005; Wang et al., 2006). Although the CRISPs were expressed in a range of 2–4% of total venom proteins in *N. kaouthia* venom samples (Laustsen et al., 2015; Tan et al., 2015d), the mRNA levels differ by 10-fold between NK-M (4.03%) and NK-T (0.32%) (Table 5). The protein expression of CRISPs also appears to undergo complex regulation, as multiple proteomic studies uncovered only the presence of natrin-type CRISP in the venoms of *N. kaouthia* from Malaysia and/or Thailand (Kulkeaw et al., 2007; Laustsen et al., 2015; Tan et al., 2015d). From the literature, the previous reported kaouthin-type CRISP was isolated and sequenced from *N. kaouthia* of an unknown geographical source (Osipov et al., 2005).

### Cobra venom factor

Cobra venom factor as a non-lethal protein resembles the complement C3 proteins structurally and functionally. Nevertheless, its pathogenic role in snake envenomation has been attributed to increasing vascular permeability and blood flow, thus facilitating venom toxins distribution (Vogel & Fritzinger, 2010). In the present study, four and two partial CVF transcripts were detected from NK-M and NK-T venom glands, respectively. Although the CVF sequences are incomplete, the assembly of these partial transcripts provides full coverage to the annotated protein (UniProtKB: Q91132) when aligned. Sequence alignment shows that the CVF transcripts from NK-T are identically matched to the CVF isolated from *N. naja siamensis* (UniProtKB: Q91132), however the *N. siamensis* could be a misnomer of *N. kaouthia* from Thailand during the time of data depositing (Data S3). While the CVF transcripts from NK-T are identical to the *N. siamensis* CVF, the sequence of CVF transcripts from NK-M on the other hand differs by 2–10% (Data S3), representing sequence variation probably associated with geographical differences of the species. Although variations were noted, it is likely that the novel CVF from NK-M is functionally similar to the annotated CVF-Q91132, as the variations detected are rather minor and not selection-driven (Data S4).

### Snake venom metalloproteinase

Snake venom metalloproteinases are proteases usually found in abundance in viper and pit viper venoms (Tan et al., 2015c; Tang et al., 2016). This multi-locus gene protein family encodes various protease subtypes that exhibit different pharmacological activities, most of which are associated with haemorrhage and coagulopathy (Fox & Serrano, 2008, 2009; Markland & Swenson, 2013). In the present study, multiple SVMP transcripts were assembled from the venom glands of *N. kaouthia*, and these transcripts encode three and seven different subtypes of SVMPs for NK-M and NK-T, respectively (Table 5). The longer transcripts from NK-M (NKM_SMP01 and NKM_SMP02) and NK-T (NKT_SMP01) were partially matched to zinc metalloproteinase-disintegrin atragin (UniProtKB: D3TTC2) of *N. atra* (Data S3). The annotated SVMPs genes in this study encode class P-III SVMPs, consistent with the finding as demonstrated at the proteomic level by multiple studies (Kulkeaw et al., 2007; Laustsen et al., 2015; Tan et al., 2015d). SVMPs presumably play a rather minor role in the pathogenesis of cobra envenomation in view of its minute content in the venom.

### Vespryn

**Figure 5:**
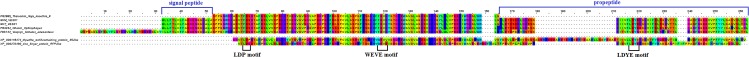
Multiple sequence alignment of vespryn (Thaicobrin) transcripts from the venom gland transcriptomes of NK-M and NK-T in comparison to vespryn sequences of representative venomous snakes. Blue, novel signal peptide/propeptide region; black, three conserved LDP, WEVE and LDYE motif of B30.2-like domains containing protein; #, indicates the toxins co-expressed in the NK-M and NK-T.

Thaicobrin is another putative toxin that has been isolated from *N. kaouthia* venom. Even though the protein was structurally characterised two decades ago, its toxic properties and pathogenic role in envenomation have not been fully investigated. The amino acid sequence available from the depository consists of only the mature chain. The present study, however, unmasked the sequences of the signal peptide and pro-peptide region (Data S3; Fig. 5). Sequencing successfully yielded a full-length vespryn transcript containing 190 amino acids from both NK-T and NK-M venom glands, with the mature chain being identically matched to the previously reported Thaicobrin (UniProtKB: P82885). Thaicobrin is highly homologous to ohanin isolated from *O. hannah* venom (Fig. 5), and it is likely that the two proteins exhibit similar pharmacological activities, i.e. inducing hyperalgesia and hypolocomotion, which may contribute to subduing of the prey (Pung et al., 2005, 2006). In contrast to ohanin which is abundant in the Malaysian king cobra venom (Tan et al., 2015a), Thaicobrin exists at a very low amount, both in terms of protein content (Tan et al., 2015d) and gene expression as shown in the present study (∼0.1% of total toxin FPKM).

### l-Amino acid oxidase

**Figure 6:**
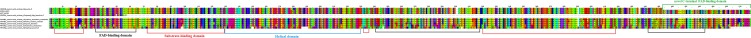
Multiple sequence alignment of l-amino acid oxidase (LAAO) transcripts from the venom gland transcriptomes of NK-M and NK-T in comparison to LAAO sequences of representative venomous snakes. Black, FAD-binding domains; red, substrate-binding domains; blue, helical domains; green, novel C-terminal FAD-binding domain; #, toxins co-expressed in the NK-M and NK-T; ^, toxin transcripts expressed in either source.

The present study detected the presence of one and eight LAAO transcripts from the venom glands of NK-M and NK-T, respectively. Among these, NKM_LAO01 and NKT_LAO01 reveal a complete sequence with 514 amino acids length (Table 5). These transcripts comprise the three well-defined domains of LAAO and are highly homologous to LAAO reported from other cobras in particular *N. atra* (UniProtKB: A8QL58) (Pawelek et al., 2000) (Data S3; Fig. 6). The present study is the first report to reveal the full sequence of *N. kaouthia* LAAO (Data S3; Fig. 6), after the previous reported LAAO for *N. naja kaouthia* specimen (unspecified origin, Japan Snake Institute) with solely N-terminal sequence (38 amino acid residues) (Sakurai et al., 2001). Multiple sequence alignment shows the snake venom LAAO sequences appear to be highly conserved across different lineages (Fig. 6) and it is noteworthy that the sequence of the major LAAO from NK-M and NK-T venom glands are completely identical (Data S3 and S4). Again, considering that the snake venom LAAO is well conserved and found in the venoms of many lineages, it is most likely evolving under strong purifying selection. LAAO transcripts constitute only about 0.1% of total toxin mRNA, and about 1% of total venom proteins (Tan et al., 2015d). This is another enzyme of low abundance and minimal mutation in most snake venoms, consistent with its rather conserved ancillary function (Du & Clemetson, 2002; Tan et al., 2015e).

### Nerve growth factor

The present study revealed a NGF transcript (complete sequence with 246 amino acid residues) in the venom glands of both NK-M and NK-T, respectively (Data S2A and S2B). The sequence is fully matched to the ‘nerve growth factor beta chain precursor’ (accession number A59218, protein information resources – PIR) isolated from *N. kaouthia* venom (Selby, Edwards & Rutter, 1987) (Data S3). This older repository was subsequently replaced by a shorter curated sequence (UniProtKB: P61899) identified from the same species with 116 amino acid residues covering only the mature chain of the NGF. To date, the role of NGF in the toxic action of snake venom remains unconfirmed. However, it has been suggested that the protein may play a role in preventing the autolysis of metalloproteinase auto-digestion as well as to exert cytotoxic and apoptosis-inducing effects (Lavin et al., 2009). Also NGF has also been shown to induce histamine release and may contribute to the hypotensive effect of the venom (Stempelj & Ferjan, 2005). In the current study, the expression of NGF genes in the venom gland of NK-T and NK-M is low and is in keeping with the minor content of this protein in the venom (<1%) (Tan et al., 2015d).

### Other minor toxin transcripts expressed in venom gland

Recent proteomics studies of *N. kaouthia* venom revealed the presence of several protein families that exist in low abundance, including KSPI, NP, PDE, 5′NUC and snaclec (Tan et al., 2015d). Results of the current *N. kaouthia* transcriptome confirmed the presence of the above-mentioned protein-encoding genes in both NK-M and NK-T venom glands (Data S2A and S2B; Table 5). All these genes were expressed at a very low level, congruent with their minor composition of the venom proteins.

**Figure 7:**
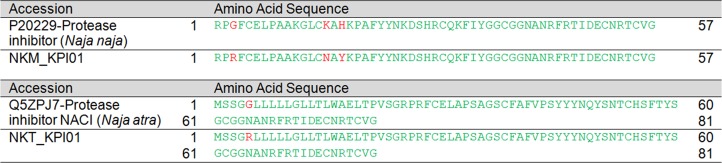
Pairwise sequence alignment of Kunitz-type serine protease inhibitor (KSPI) transcripts from the venom gland transcriptomes of NK-M and NK-T in comparison to the annotated KSPI sequences. Green, consensus sequence; red, sequence diversification; black, mismatched sequences.

Two and three KSPI transcripts (all with full sequence) were reported in the venom glands of NK-M and NK-T, respectively. Of these, one gene was co-expressed in NK-M and NK-T, and their sequences were aligned for comparison in Data S3. The most abundantly expressed KSPI isoforms in the venom gland of NK-M (NKM_KPI01) and NK-T (NKT_KPI01) is found to be different (Fig. 7). The respective sequences of NK-M and NK-T are highly homologous to serine protease inhibitors reported from other cobra species: *N. naja* (UniProtKB: P20229) for NK-M transcript, and *N. atra* (UniProtKB: Q5ZPJ7) for NK-T transcript (Shafqat et al., 1990; Zhou et al., 2004) (Fig. 7).

**Figure 8:**
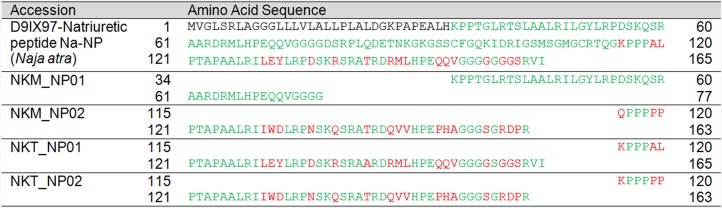
Pairwise sequence alignment of natriuretic peptide (NP) transcripts from the venom gland transcriptomes of NK-M and NK-T in comparison to the annotated NP sequences. Green, consensus sequence; red, sequence diversification; black, mismatched sequences.

In this study, partial NP transcripts were also identified and annotated to NPs reported from *N. atra* (UniProtKB: D9IX97) (Fig. 8). The venom-derived NP has been shown to induce rapid relaxation of phenylephrine-precontracted rat aortic strips, and to stimulate cGMP production, inducing hypotension in experimental rats (Zhang et al., 2011). It has been reported that most of the NPs found in elapid venoms are of the atrial-NP (ANP), or B-type NP (BNP) which do not have the bradykinin-potentiating peptide (BPP) domain and the part of the linker sequence (Zhang et al., 2011). This is largely consistent with the major NP transcripts found in this study and the ANP and BNP detected at the protein level of *N. kaouthia* venom (Tan et al., 2015d). The present study also detected PDE transcripts in NK-M and NK-T venom glands at very low level of gene expression (<0.01%). Among these, one PDE subtype was co-expressed (also most highly expressed) in both NK-M and NK-T venom glands, and exhibits full-length sequence coverage (NKM_PDE01 and NKT_PDE01) (Data S2A and S2B; Table 5). The two full-length protein-encoding transcripts were identical (Data S3), and are highly homologous to the annotated sequence reported for *M. fulvius* (UniProtKB: U3FAB3). The pathogenic role of PDE may be shaped towards the potentiation of venom-induced hypotension and paralysis through the release of purines (Aird, 2002, 2005; Dhananjaya & D’Souza, 2010).

Snake venom snaclecs are typical toxins present in viper or pit viper venoms (Arlinghaus & Eble, 2012; Du & Clemetson, 2009). Recent venomic studies showed that snaclecs are not exclusive to viperid venoms—for instance, the presence of snaclecs as a minor protein component has been shown in *N. kaouthia* venom (Laustsen et al., 2015; Tan et al., 2015d) and this is well supported by their gene expression in this present study. A total of four different snaclecs subtypes were detected in both NK-M and NK-T venom glands, with NKM_SCL01 and NKT_SCL01 being the most highly expressed (<0.3%) (Table 5). The two snaclec transcripts were identical, with sequence homologous to C-type lectins-BFL-1 (UniProtKB: Q90WI8) of *Bungarus fasciatus* (Data S3). Snaclecs are considered ‘haemotoxic’ toxins for their ability to disrupt platelet functioning (Clemetson, 2010); this effect is however less important in *N. kaouthia* envenoming in this region as thrombocytopaenia attributable to its bite has never been reported.

### Novel transcripts detected only at transcriptomic level

The transcriptomic study further identified 11 putative toxin families comprising a total of 16 and 16 toxin transcripts, respectively, in the venom gland of NK-M and NK-T. These include cystatin, SVSP, AChE, HYA, CF, PLB, waprin, neprilysin, AP, DPP-IV and IGF. Most of these toxin families have not been reported in cobra venom, presumably due to their very low level of protein expression; while at the transcriptional level, only a single cystatin transcript of *N. kaouthia* (Thailand) has been reported from an earlier cloning study (Richards et al., 2011). The cystatin transcript, deposited as UniProtKB: E3P6P4, is identical to transcripts NKM_CYS02 and NKT_CYS02 uncovered in this study (Data S3). Among the four additional cystatin-like transcripts sequenced in the current study, three of which were co-expressed in NK-M and NK-T (Data S2A, S2B and S3; Table 5). Of note, NKM_CYS01 and NKT_CYS01 from NK-M and NK-T, respectively, are the most highly expressed cystatin transcripts and possess novel sequence that is unique from the annotated protein (Data S3). Although the actual role of cystatin remains equivocal, it was suggested that its protease inhibition properties may contribute to the stability of toxin proteins in the venom glands (Richards et al., 2011).

**Figure 9:**
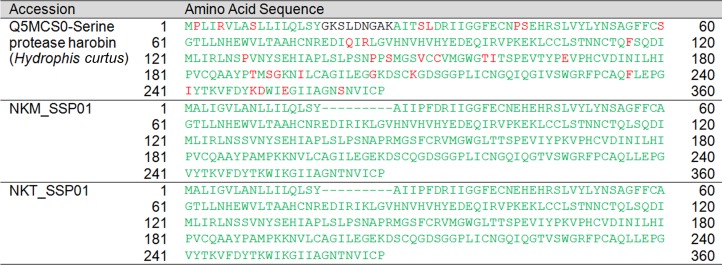
Pairwise sequence alignment of snake venom serine protease (SVSP) transcripts from the venom gland transcriptomes of NK-M and NK-T in comparison to the annotated SVSP sequences. Green, consensus sequence; red, sequence diversification; black, mismatched sequences.

Snake venom serine proteases are generally involved in venom-induced consumptive coagulopathy (Serrano & Maroun, 2005). There is one full-length protein-encoding SVSP transcript detected in both NK-M and NK-T venom glands (transcripts NKM_SSP01 and NKT_SSP01). Both of the transcript from NK-M and NK-T are highly homologous to the SVSP-harobin (UniProtKB: Q5MCS0) from *Hydrophis curtus* (Fig. 9). However, none of the proteases were detected in the venom proteome (Tan et al., 2015d), indicating that the proteins were either not translated or exist at very low level, consistent with the negative finding of coagulopathy in *N. kaouthia* envenomation.

**Figure 10:**
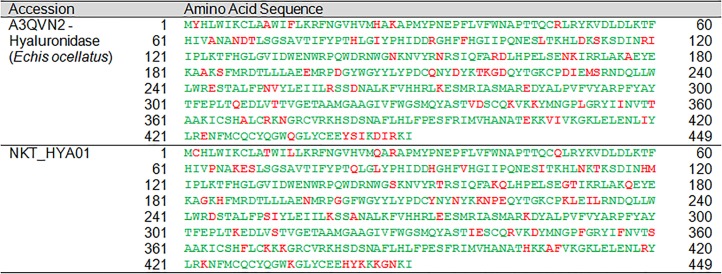
Pairwise sequence alignment of hyaluronidase transcript from the venom gland transcriptome of NK-T in comparison to the annotated hyaluronidase sequence. Green, consensus sequence; red, sequence diversification; black, mismatched sequences.

Acetylcholinesterase (Ahmed et al., 2009) and HYA (Tu & Hendon, 1983) are two well-investigated snake venom enzymes, yet their content in snake venom is usually very low and often undetectable in venom proteome, although the enzymatic activities have been detected in various cobra venoms (Tan & Tan, 1988). In the present study, transcripts of the two enzymes were reported from *N. kaouthia* venom glands, supporting the expression of the proteins in *N. kaouthia* venom. Importantly, the full sequences of AChE were unveiled in the venom gland transcriptomes of NK-M (NKM_ACE01) and NK-T (NKT_ACE01), with identical sequences observed (Data S3). This is also the first report of *N. kaouthia* AChE sequence (as well as the complete sequence in *Naja* species), which are homologous to the AChE (UniProtKB: Q92035) reported from *B. fasciatus* (Data S3). On the other hand, HYA gene expression was detected in NK-T venom gland, with full-length protein-encoding transcript (NKT_HYA01) obtained. The HYA transcript (NKT_HYA01) representing the first reported sequences of HYA of *N. kaouthia* as well as in *Naja* species and share 84% homology with HYA from *Echis ocellatus* (UniProtKB: A3QVN2) (Fig. 10). HYA gene, a relatively less investigated venom enzyme family, is likely well-conserved across many lineages of Elapidae and Viperidae as a result of purifying selection.

It is interesting to note that the CF X transcript was also detected (albeit at very low abundance) in *N. kaouthia* venom gland transcriptomes, even though *N. kaouthia* venom is not known to exhibit procoagulant activity. Another protein family of the putative toxin is PLB; this is an ‘emerging’ snake venom enzyme with little characterisation, only detected recently in many snake venoms through the high-resolution mass spectrometry technique. Complete sequence for PLB from both NK-M and NK-T venom glands was also obtained in the present study. The transcripts (NKM_PLB01 and NKT_PLB01) with higher expression show sequences that are highly homologous to PLB-81b isolated from *Drysdalia coronoides* (Chatrath et al., 2011) (Data S3). This is also the first report of PLB sequence in *Naja* species though the activity has been reported in *N. naja* venom (Shiloah, 1974).

**Figure 11:**
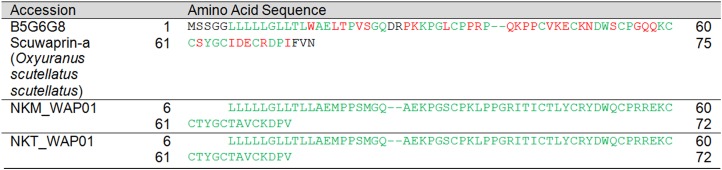
Pairwise sequence alignment of waprin transcripts from the venom gland transcriptomes of NK-M and NK-T in comparison to the annotated waprin sequence. Green, consensus sequence; red, sequence diversification; black, mismatched sequences.

Waprins with little known toxic properties have not been extensively reported. The current study first reported the detection of waprins at the transcriptional level in *Naja* species (one transcripts in NK-M and NK-T venom glands, FPKM < 0.01%). The transcripts, with full amino acid sequences, were annotated to Scuwaprin-a (*Oxyuranus scutellatus scutellatus*) (Data S3; Fig. 11). Neprilysin-like protein is a novel metalloendopeptidase that has a wide range of functional targets in the regulatory processes of natriuretic and vasodilatory neuropeptides (Turner, Isaac & Coates, 2001). It has been reported as putative toxins in the saw-scaled viper and king cobra (Casewell et al., 2009; Tan et al., 2015a; Vonk et al., 2013), though its role in the pathogenesis of envenomation has not been established. In this study, neprilysin-like transcripts were detected in both venom glands. The transcripts are identical between the both samples, while the one from NK-T shows a full sequence. This represents a novel full amino acid sequence of neprilysin-like protein in *Naja* species, which shares a high degree of homology to the neprilysin identified from *Python bivittatus* (Data S3; Table 5). The finding may reflect the presence of venom gene orthologues in the ancestral genome prior to the expansion and diversification of various snake lineages (Reyes-Velasco et al., 2015).

The present study also showed the presence of several transcripts encoding APs (two subtypes), dipeptidylpepidase-IV (one transcript each sample, with full sequence) and IGFs (one transcript each sample, with full sequences). These can at best be considered as putative toxins as the functionalities of these proteins have not been rigorously established. APs are exo-metalloproteases that function in the physiological maintenance of the blood pressure (Vaiyapuri et al., 2010), while DPP-IV is a highly glycosylated serine protease that may counteract the hypertensive response in the envenomed prey by destroying hypertensive peptidyl hormones (Aird, 2008). The level of expression of these three putative toxins was very low (<0.03%) and the expressed proteins were not detectable even with the use of highly sensitive nano-LCMS/MS technique (Tan et al., 2015d), although some authors reported the presence of AP protein in certain snake venoms (Faiz et al., 1996; Gasparello-Clemente & Silveira, 2002). For *N. kaouthia*, full sequences of DPP-IV and IGF were available from the present study and these are the only ones reported from the venom gland transcriptomes of *Naja* species thus far.

## Conclusion

This study set to elucidate the venom gland transcriptomes of *N. kaouthia* from two different geographical origins: Malaysia and Thailand. The findings demonstrated the unique expression patterns of toxin-encoding gene transcripts with high redundancy in comparison to the non-toxin genes. A total of 22 venom gene families were co-expressed in both NK-M and NK-T venom glands (out of the 24 families identified), with 15 gene families reported for the first time in *N. kaouthia* at transcriptional level. Comparatively, the expressions of venom genes in NK-M and NK-T were generally comparable while being dominated by 3FTxs. Inter-locale variations were remarkable in the magnitude of gene expression, for example, NK-T transcriptome is dominated by LNTX expression, whereas NK-M is dominated by SNTX; CTX-3 was exclusively expressed in NK-T, whereas CTX-5 is the most abundantly expressed CTX gene in NK-M. Despite the variation of the toxin subtype expressed between the both NK-M and NK-T, the findings support the role of particular gene expression pattern in moulding the venom repertoire, possibly driven mainly by strong purifying selection within the same population although the variations appeared not supported by selection analysis in the current study. Also the study has greatly enriched the venom sequence database for *Naja* species and revealed the complete amino acid sequence of more than 30 venom proteins, many of which are novel having not been reported previously. Together, the results will contribute to better understanding the biological and clinical implications of *N. kaouthia* venom variations in this region.

## Supplemental Information

10.7717/peerj.3142/supp-1 Supplemental Information 1 Clustering of sequence reads of the venom gland transcriptomes of both *Naja kaouthia* sourced from Malaysia (NK-M) and Thailand (NK-T).

10.7717/peerj.3142/supp-2 Supplemental Information 2 Classification and detailing of toxin transcripts from the venom gland transcriptome of Malaysian *Naja kaouthia* (NK-M).

10.7717/peerj.3142/supp-3 Supplemental Information 3 Classification and detailing of toxin transcripts from the venom gland transcriptome of Thai *Naja kaouthia* (NK-T).

10.7717/peerj.3142/supp-4 Supplemental Information 4 Comparison of toxin transcript sequences from the venom gland transcriptomes of Malaysian (NK-M) and Thai *Naja kaouthia* (NK-T) with the Nr-annotated toxins deposited in public database.

10.7717/peerj.3142/supp-5 Supplemental Information 5 Comparison and substitution analyses of nucleotide sequences from the venom gland transcriptomes of Malaysian (NK-M) and Thai *Naja kaouthia* (NK-T). First column indicates protein families, accession number, ID and species of the annotated protein as detailed in Data S3. Second column shows set of sequence comparisons that have been carried out. Third column indicates protein coding ID of the current study. Forth column shows line number in which the peptide matched to the annotated protein. Fifth column shows nucleotide sequence comparison of both NK-M and NK-T transcripts reported in the current study. Sixth column shows percentage of similarity between the both NK-M and NK-T nucleotide sequence. Column seventh to thirteen show values of substitution, synonymous-substitution, nonsynonymous-substitution, Ka, Ks, KaKs and significance value (P, using Fisher’s exact test) when comparing nucleotide sequences from both NK-M and NK-T using “Approximate method.” Green indicates peptide matched; red indicates amino acids diverged from the aligned sequences; black indicates mismatched sequences. *Nucleotide sequence comparison was based on the co-expressed transcripts from the Supplemental Data S3, in which only consensus regions were used in substitution rates calculation.

## List of Abbreviations

NK-M: Malaysian *N. kaouthia*
NK-T: Thailand *N. kaouthia*
3FTx: three-finger toxin
L: long chain
S: short chain
NC: non-conventional
CRISP: cysteine-rich secretory protein
CVF: cobra venom factor
PLA_2_: phospholipase A_2_
SVMP: snake venom metalloproteinase
NGF: nerve growth factor
KSPI: Kunitz-type serine proteinase inhibitor
NP: natriuretic peptide
snaclec: C-type lectin/lectin-like protein
LAAO: l-amino acid oxidase
PDE: phosphodiesterase
AP: aminopeptidase
SVSP: snake venom serine protease
PLB: phospholipase-B
5′NUC: 5′nucleotidase
DPP-IV: dipeptidylpeptidase-IV
CF: coagulation factor
AChE: acetylcholinesterase
IGF: insulin-like growth factor
FPKM: fragments per kilobase of exon model per million mapped reads.

## Additional Information and Declarations

### Field Study Permissions

The following information was supplied relating to field study approvals (i.e. approving body and any reference numbers): The works were approved by the Department of Wildlife and National Parks of Peninsular Malaysia (#JPHL&TN (lP):80-4/2) and the Institutional Animal Use and Care Committee (IACUC) of the University of Malaya (#2013-11-12/PHAR/R/TCH).

### cDNA Deposition

The following information was supplied regarding the deposition of cDNA sequences: Sequence data from the venom gland transcriptomes of *N. kaouthia* from Malaysia (NK-M) and Thailand (NK-T) have been deposited in National Centre for Biotechnology Information (NCBI) Sequence Read Achieve under Bioproject: PRJNA302200 (AC ID: SRP066203) (NK-M: SRR2917658; NK-T: SRR2917657) (http://www.ncbi.nlm.nih.gov/sra/SRP066203).

### Data Availability

The following information was supplied regarding data availability: National Centre for Biotechnology Information (NCBI) Sequence Read Achieve: PRJNA302200 (AC ID: SRP066203) (NK-M: SRR2917658; NK-T: SRR2917657). https://www.ncbi.nlm.nih.gov/sra/SRX1432814[accn]. https://www.ncbi.nlm.nih.gov/sra/SRX1432812[accn].
